# Biochemistry and genetics of folate metabolism

**DOI:** 10.1186/1743-8454-7-S1-S4

**Published:** 2010-12-15

**Authors:** Rima Rozen

**Affiliations:** 1 James McGill Professor of Human Genetics and Pediatrics, McGill University, Montreal, Canada

## 

Folates serve in many critical one-carbon transfer reactions including nucleotide synthesis, amino acid synthesis and methylation. Methylation is important for gene expression (through methylation of DNA), neurotransmitter synthesis and maintenance of homocysteine (a potentially toxic amino acid) at low levels. Genetic and nutritional deficiencies in folate metabolism may modulate risk for several complex traits including neural tube defects (NTD) and other birth defects, cardiovascular disease, pregnancy complications and some cancers. Inadequate dietary folate has been recognized as a risk factor in approximately 2/3 of NTD although metabolic disturbances in folate metabolism have emerged more recently as contributors to this common multifactorial disorder. Fortification of food with folate has been established in many countries to reduce the incidence of NTD; although NTD rates have in fact decreased in those countries, there are controversies regarding the impact of food fortification on other folate-related disorders. The first genetic risk factor identified for NTD is a common variant in methylenetetrahydrofolate reductase (MTHFR), the enzyme that synthesizes the primary circulatory form of folate, 5-methyltetrahydrofolate, which is utilized in the remethylation of homocysteine to methionine. This variant, present in the homozygous state in approx. 10% of many European and North American populations, is responsible for a fraction of the folate-responsive NTDs. Other variants in the pathway have been identified and may contribute although additional studies are required. A mouse model for MTHFR deficiency has been useful in studying the biochemical disturbances and mechanisms that could contribute to NTD and other complex traits. The presentation will focus on the impact of genetic polymorphisms/relevant enzymes on NTD risk, the use of mouse models to study mechanisms, and some aspects of the controversy surrounding high folate intake post-fortification.

